# Sustained Reduction of Ventilator-Associated Pneumonia Through a Structured Interdisciplinary Quality Improvement Strategy

**DOI:** 10.7759/cureus.115363

**Published:** 2026-08-28

**Authors:** Cindy L Austin, Liz Day, Brian Draper

**Affiliations:** 1 Trauma Research, Mercy Health, Springfield, USA; 2 Trauma Program, Mercy Health, Springfield, USA; 3 General and Trauma Surgery, Mercy Health, Springfield, USA

**Keywords:** burn, critical care, intensive care unit, mechanically ventilated patient, quality improvement, trauma, ventilator-associated pneumonia

## Abstract

Ventilator-associated pneumonia (VAP) is a source of morbidity and mortality among critical care patients, particularly in trauma and burn. In 2022, our hospital identified a high incidence of VAP. This prompted a comprehensive evaluation and implementation of targeted interventions to improve outcomes in ventilated patients. The objective of this study was to evaluate the impact of a multifaceted strategy for VAP prevention by assessing its effectiveness in reducing VAP incidence through consistent application of evidence-based interventions.

This pre- and post-quality improvement study was conducted at a Midwest Level I trauma center from 2022 to 2025. Interventions included standardized documentation with validation by a designated trauma program member, consistent use of a respiratory care protocol for intubated patients, and emphasis on extubation or tracheostomy decision-making during daily multidisciplinary rounds. Nursing staff introduced structured care cards to monitor oral hygiene, prophylaxis, sedation breaks, and equipment assessments.

Audits were conducted using a visual accountability board system to reinforce compliance and enable immediate corrective actions. A Ventilator-Associated Event Committee representing nursing, infection prevention, respiratory therapy, trauma, critical care medicine, and quality improvement met monthly to review data, refine interventions, and support ongoing staff education. The results showed adherence to evidence-based VAP prevention practices improved, with a sustained reduction in VAP incidence from 3.3% to 0.4% across reporting periods. Coordinated interdisciplinary collaboration efforts, guided by structured and data-driven strategies, resulted in significant reductions in VAP rates and improved the overall quality of care for mechanically ventilated patients.

## Introduction

Prevalence

Given the highly compromising and vulnerable nature of the trauma, burn, and critical care patient population, the risk for developing ventilator-associated pneumonia (VAP) is elevated. VAP remains one of the most significant yet preventable complications affecting mechanically ventilated patients in critical care environments.

Recent evidence indicates that VAP affects a substantial proportion of critically ill patients, with reported rates ranging up to approximately 30% among those receiving mechanical ventilation [1]. VAP is one of the leading forms of hospital-acquired infections, contributing to increased morbidity, prolonging mechanical ventilation, extending intensive care unit (ICU) and hospital length of stay, and driving up healthcare costs. Although VAP rates have reportedly decreased over the past 20 years, approximately 5%-10% of mechanically ventilated patients are still treated for VAP or associated events [2].

Strategies and bundles

Multiple strategies have been implemented to decrease this risk, including VAP prevention bundles, enhanced oral care, elevation of the head of the bed, subglottic suctioning, and staff education [1,3,4]. Recent systematic reviews and large epidemiologic datasets highlight that evidence-based prevention bundles, such as those endorsed by the Institute for Healthcare Improvement (IHI), significantly reduce VAP incidence when consistently implemented. Also, these bundles commonly include optimized sedation and weaning protocols, endotracheal tube cuff-pressure management, and daily assessments for extubation readiness [4,5]. Evidence suggests that early intubation within eight hours of admission and bronchoalveolar lavage within 24 hours can significantly reduce VAP incidence in trauma patients [6].

Lack of compliance

Despite the adoption of these interventions, preventing VAP remains a challenge due to the need for ongoing vigilance, adherence to best practices, and low real-world compliance with prevention bundles. Studies report that adherence to ventilator care bundles varies widely across institutions and providers, with full compliance reported in <1% of patients in some cohorts. Importantly, each additional bundle element performed is associated with meaningful reductions in VAP risk, reinforcing that reliable, multidisciplinary execution of these practices is essential for improving outcomes [7].

Structured approach to VAP and gap

Investigations from diverse international settings show that the most effective VAP reduction programs rely on standardized documentation, structured education, and continuous monitoring of bundle adherence [8]. Multidisciplinary structures are increasingly recognized as necessary to improve VAP prevention reliability. Guidelines recommend coordination among nursing, respiratory therapy, infection prevention, physicians, and quality improvement (QI) teams to ensure accurate surveillance and interpretation of ventilator-associated events (VAEs). Furthermore, adequate education helps staff to follow prevention strategies.

Recent research shows ICU clinicians still have knowledge gaps, demonstrating the need for structured and ongoing educational programs to support sustained improvements [2,4,9,10]. Baseline assessment at our hospital revealed a high incidence of VAP rates, placing the trauma program in the 10th decile for VAP incidence among ventilated patients according to Trauma Quality Improvement Program (TQIP) data [11]. This finding prompted a comprehensive review of surveillance practices, protocol adherence, and bedside clinical care. A multifaceted, team-based QI initiative was developed to reduce VAP incidence based on structured monitoring and enhanced accountability. This study aims to evaluate the impact of a multifaceted strategy to enhance VAP prevention by assessing its effectiveness in reducing VAP incidence through consistent implementation of evidence-based interventions.

## Materials and methods

A prospective QI initiative was conducted at a Midwest Level I tertiary care hospital (Mercy Hospital, Springfield, MO, USA) from 2022 to 2025. The study aimed to reduce VAP among mechanically ventilated adult patients through implementation of a multifaceted prevention strategy. This project was conducted as a QI initiative and, in accordance with institutional policy, was not subject to Institutional Review Board review.

Study population

Adult patients aged ≥18 years admitted to ICUs who required invasive mechanical ventilation for at least 48 hours were included. Patients were excluded if they had pneumonia present on admission or developing within 48 hours of intubation, were transferred from outside facilities with a pre-existing diagnosis of VAP, were placed on comfort care, were extubated within 48 hours, or had incomplete clinical or surveillance data. Consecutive sampling was used, including all eligible patients during the three-year study period. Potential confounding variables included patient comorbidities, severity of illness, duration of mechanical ventilation, ICU type, seasonal variation, and staffing patterns. Standardized protocols were implemented to minimize variability across units. Observed VAP counts were compared to expected values from the TQIP using observed-to-expected (O/E) ratios. Poisson-based methods were used to account for rare events, and trends over time were evaluated using Poisson regression. A p-value < 0.05 was considered significant.

Initiatives implemented

A Ventilator-Associated Event Committee was established consisting of representatives from nursing, infectious disease and prevention, respiratory therapy, trauma, critical care medicine, and QI. The QI bundle included standardized documentation and validation, in which a designated trauma program member validated all VAP surveillance entries to ensure accuracy. Providers were guided to consistently utilize a respiratory care protocol for intubated patients. A daily rounding tool was implemented across all ICUs to ensure that the daily multidisciplinary team addressed timely decisions regarding extubation or tracheostomy, with real-time reporting of protocol compliance (see Appendix A). The nursing staff introduced structured care cards (K-card) to monitor oral hygiene, prophylaxis, sedation breaks, and equipment assessments (see Appendix B). The K-card was used to audit and reinforce compliance, enabling real-time identification of deviations in care and feedback that supported immediate corrective action. Monthly meetings facilitated data analysis, evaluation of interventions, and ongoing staff education. Data analysis of outcome and process measures included tracking the primary outcome: VAP incidence (defined as the percentage of ventilated patients with VAP within a reporting period), as reported to TQIP and monitored internally. 

## Results

**Figure 1:**
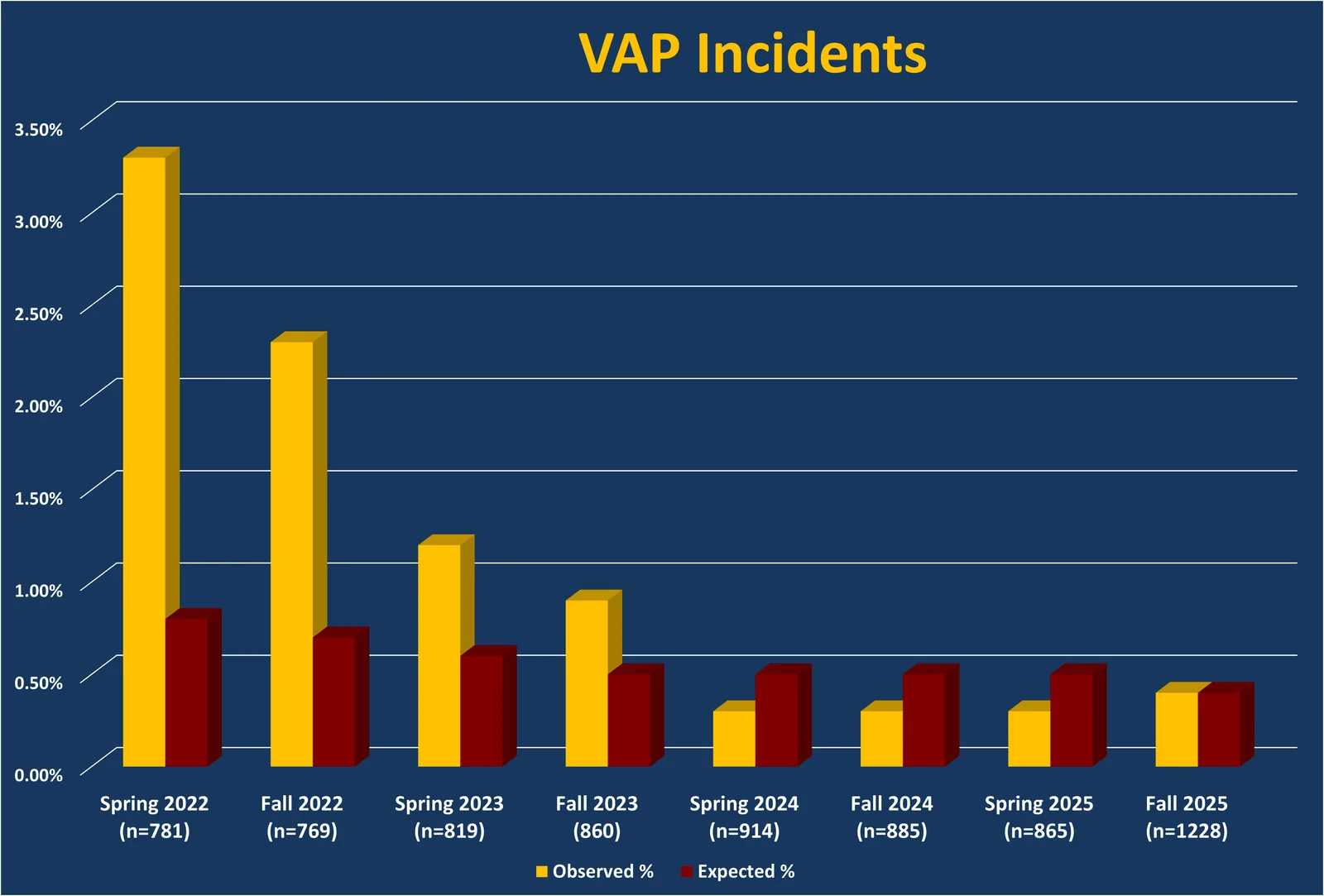
Impact of VAP Initiatives A consistent decline in the percentage of patients developing VAP-from 3.3% to 0.4% across a 3-year reporting period. VAP: ventilator-associated pneumonia

Analysis of outcome and process measures across reporting periods demonstrated significant reductions in VAP following implementation of multidisciplinary daily rounds, K-cards, and enhanced tracking processes. A total of 7,121 mechanically ventilated patients were included across all reporting periods. Observed VAP rates decreased steadily from 3.3% (26/781; 95% CI, 2.1%-4.6%) in Spring 2022 to 0.4% (5/1,228; 95% CI, 0.05%-0.76%) in Fall 2025, representing a statistically significant decline (p < 0.05). In early reporting periods, observed VAP rates were consistently above the TQIP expected values (O/E ratios > 1); however, from Spring 2024, observed rates met or fell below expected benchmarks. This reflects a sustained reduction in VAP incidence over time. Initially, the ICU ranked in the 10th decile for VAP incidence, but post-intervention, there was a consistent decrease in the proportion of patients developing VAP-from 3.3% to 0.4% over the three-year reporting period (Figure 1).

## Discussion

Our results align with literature showing that ventilator care bundles, similar to our multifaceted strategy combining comprehensive evaluation with targeted interventions, lead to clinically important reductions in VAP, particularly when adherence is high and sustained. Systematic reviews including 22 studies report typical reductions exceeding 36%, with four studies approaching near-zero rates following robust implementation and VAP monitoring. Observational analyses further indicate that each additional bundle element performed is associated with lower odds of VAP, highlighting the importance of complete bundle delivery [4,7].

Our approach also reflects QI work demonstrating that education, checklists, and audit-and-feedback processes drive bundle reliability and correlate with outcome improvement. Reports from diverse healthcare settings have documented sustained >90%-95% compliance and achieved step-wise reductions in VAP after implementing visual management systems and defect-recovery processes similar to our accountability process [8,12-14].

Mechanisms and implementation insights

The impact observed in our program can be attributed to multiple reinforcing mechanisms. The establishment of the VAE Committee enhanced surveillance, data transparency, and feedback, while nurse-driven oral care checks-supported by structured K-cards and consistent use of the daily rounding tool-enhanced accountability and process reliability. The key strength was the interdisciplinary design of the initiative, integrating nursing, respiratory therapy, infection disease and prevention, trauma and critical care, and QI, reflecting established best-practice recommendations [4]. Additionally, system-level standardization of documentation workflows and bundle practices through QI processes promoted sustained adherence and enabled timely correction of care gaps [8]. Ongoing education and competency reinforcement further supported these efforts, consistent with evidence demonstrating that provider knowledge and training are key factors in influencing compliance with infection prevention practices [9]. Together, these elements created a cohesive, high-reliability framework that facilitated durable improvements in VAP prevention and patient outcomes.

Limitations

The analysis used an unadjusted Poisson regression model, and confounders-including patient comorbidities, severity of illness, duration of mechanical ventilation, ICU type, seasonal variation, and staffing patterns-were not controlled for, which may introduce bias and limit causal inference. Additionally, as a single-center observational QI study, the findings may have limited generalizability.

## Conclusions

Collaborative interdisciplinary efforts, supported by structured and data-informed strategies, led to significant reductions in VAP rates and improved the overall quality of care for mechanically ventilated patients. This model demonstrates the effectiveness of systematic, team-based approaches in reducing ventilator-associated complications and offers a framework that may be adapted across other critical care settings.

## Figures and Tables

**Figure 2:**
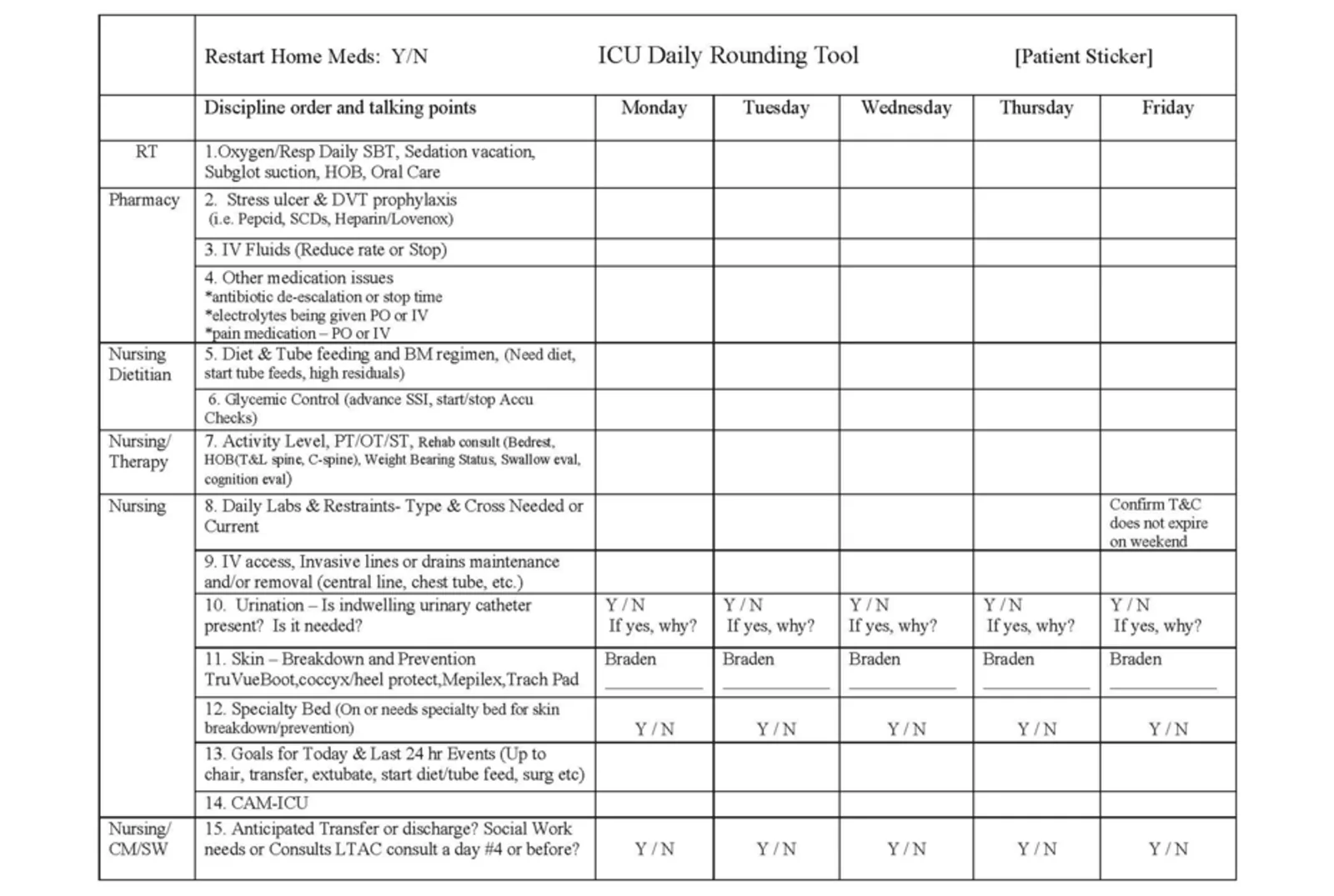
Daily Rounding Tool The daily rounding tool was implemented to ensure the multidisciplinary team addressed timely decisions regarding extubation or tracheostomy care with real-time reporting of protocol compliance.

**Figure 3:**
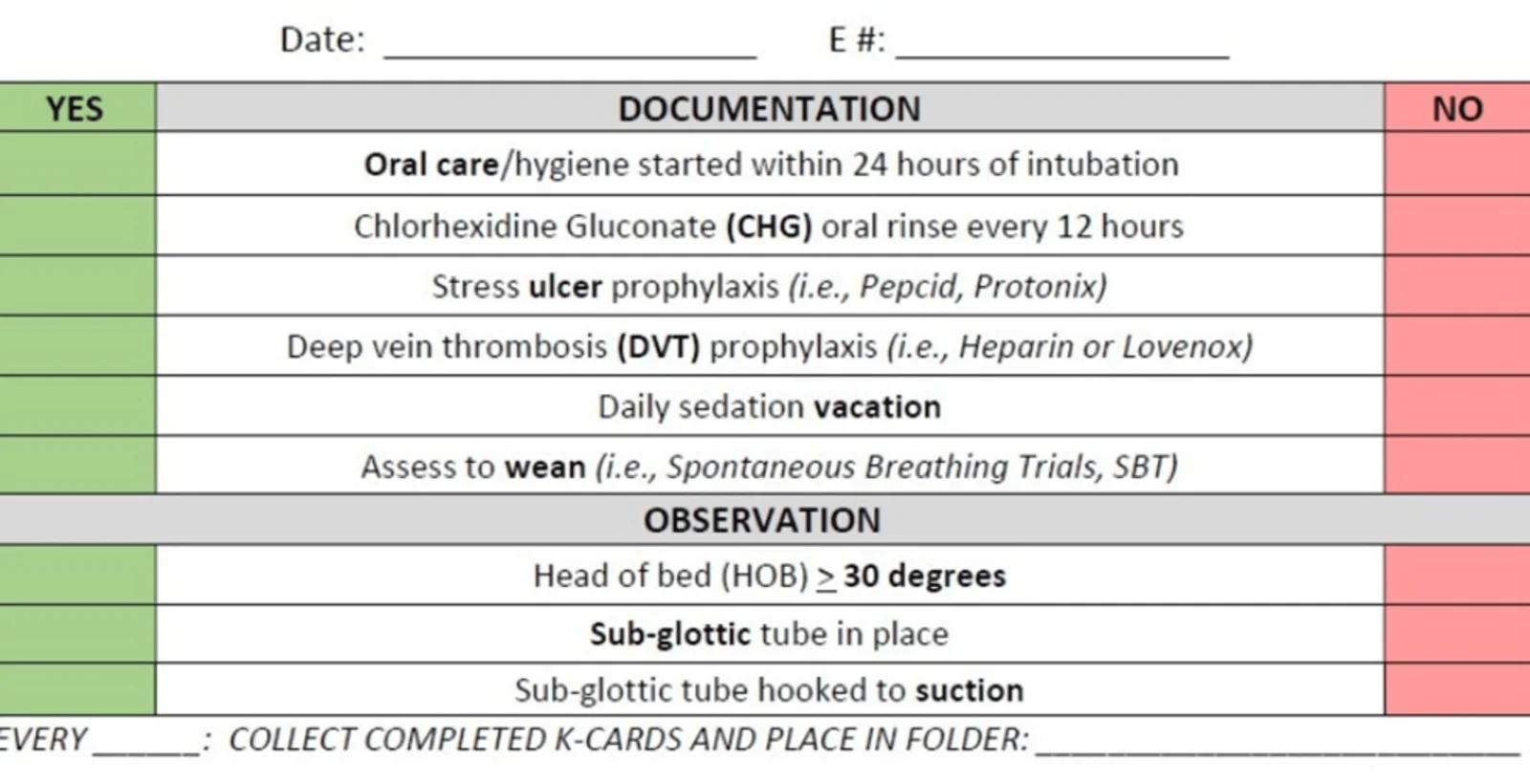
K-Card K-card implemented to monitor key care elements, audit compliance, and enable real-time identification of issues with immediate corrective feedback.

## Appendices

Appendix A 

Appendix B
